# Gene expression profiling using formalin-fixed paraffin-embedded primary specimens of AIDS-related Lymphomas

**DOI:** 10.1186/1750-9378-7-S1-O21

**Published:** 2012-04-19

**Authors:** Duane Hassane, Jessica Padilla, Rob Kim, Lisa Giulino, Susan Matthew, Wayne Tam, Amy Chadburn, Sharon Barouk, Clement Adebamowo, Lorenzo Leoncini, Tanuja Shet, Mark A Rubin, Ethel Cesarman

**Affiliations:** 1 Department of Pathology, Weill Cornell Medical College, New York, NY, USA; 2 Northwestern University-Feinberg School of Medicine, Chicago, IL, USA; 3 University of Ibadan, Nigeria; 4 Department of Human Pathology and Oncology, University of Siena, Siena, Italy; 5 Tata Memorial Hospital, Mumbai, India

## Background

Gene expression profiling has been useful for classification and prognostication of a variety of hematologic neoplasms occurring in the general population. This type of analysis of AIDS related lymphomas (ARLs) has been limited because of their rarity, heterogeneity and lack of frozen tissue for analysis, with the largest studies including 25 cases (Klein et al., Blood 2003, Deffenbacher et al., J AIDS 2010). To overcome this limitation, we employed a *cDNA-based microarray technology*, *DNA-mediated Annealing*, *Selection*, *Ligation and Extension* (*DASL*) *for the analysis of* formalin-fixed paraffin-embedded (FFPE) *archival samples*, allowing us to perform gene expression analysis of the largest cohort of ARLs thus far.

## Material and methods

We performed expression profiling from FFPE samples of AIDS related lymphoma for using DASL (Illumina^®^), with modification of the cDNA and quality control (QC) steps. The following cases of ARL with confirmed diagnosis and sufficient RNA were used for evaluation in duplicate: Weill Cornell Medical College in New York, USA (36 cases), the AIDS Malignancy Consortium (AMC) (24 cases), University of Siena, Italy (20 cases), Italy (21 cases), Tata Memorial Hospital in Mumbai, India (35 cases), University of Ibadan, Nigeria (1 case). Non-AIDS lymphomas were included as controls (15 cases from India and 13 cases from Weill Cornell). A 1mm diameter core was obtained from each block and RNA extracted. Tissue microarrays were also prepared of the available specimens, and characterization of viral status and lymphoma subtype were determined by immunohistochemistry and *in situ* hybridization for Epstein-Barr encoded RNA (EBER). Fluorescent in situ hybridization (FISH) was used to evaluate for genomic deletions in A20, and translocations of cMYC, BCL-2 and BCL-6.

## Results

Gene expression profiling of 126 cases was initially performed using DASL. Quality control assessment and data analysis revealed poor predictive ability of the QC method and poor quality of the cDNA resulting in data variability and lack of reproducibility. Therefore, we developed alternative methodologies for cDNA preparation and assessment of quality of the RNA, resulting in more than double the number of genes detected and good reproducibility in the majority of the samples. Analysis of gene expression profiling of 116 cases of ARL and 28 matched non-AIDS lymphomas will be presented.

## Conclusions

We have developed methods that allow gene expression analysis of large numbers of ARLs, which will pave the way of determining whether subtype specific signatures resemble those of lymphomas in immunocompetent individuals, and eventually if these have clinical implications.

